# Profil épidémiologique, aspects diagnostiques et évolutifs des patients tuberculeux au centre de diagnostic de la tuberculose et des maladies respiratoires de Settat, Maroc

**DOI:** 10.11604/pamj.2022.42.185.35250

**Published:** 2022-07-07

**Authors:** Mohamed Chahboune, Mohamed Barkaoui, Younes Iderdar, Nadia Alwachami, Yassmine Mourajid, Mohamed Ifleh, Karima Boumendil, Khadija Bachar, Saad El Madani

**Affiliations:** 1 Hassan First University of Settat, Higher Institute of Health Sciences, Laboratory of Health Sciences and Technologies, Settat, 26000, Morocco,; 2 Hassan First University of Settat, Higher Institute of Sport, Laboratory of Health Sciences and Technologies, Settat, 26000, Morocco,; 3 Université Mohammad V, Faculté de Médecine et de Pharmacie, Laboratoire d´Hématologie, Rabat, Maroc,; 4 Centre de Diagnostic de la Tuberculose et des Maladies Respiratoires, Settat, 26000, Maroc,; 5 Higher Institute of Health Sciences, Laboratory of Health Sciences and Technologies, Settat, 26000, Morocco

**Keywords:** Tuberculose, guérison, diagnostic, facteurs associés, Tuberculosis, recovery, diagnosis, associated factors

## Abstract

**Introduction:**

la tuberculose demeure un problème de santé publique au Maroc. Ce travail vise à étudier le profil épidémiologique, les aspects évolutifs et diagnostiques de la tuberculose à Settat, Maroc.

**Méthodes:**

il s´agit d´une étude rétrospective descriptive et analytique, étalée sur la période allant du 1^er^ janvier 2015 au 31 décembre 2019, réalisée par l´exploitation des dossiers d´une cohorte de patients tuberculeux pris en charge au centre de diagnostic de la tuberculose et des maladies respiratoires de Settat au Maroc.

**Résultats:**

nous avons répertorié un total de 1270 cas de tuberculose. L´atteinte pulmonaire était plus fréquente que l´atteinte extrapulmonaire. La tuberculose ganglionnaire était prédominante parmi les cas de tuberculose extrapulmonaire. Les formes graves étaient représentées par 10 cas de méningite tuberculeuse et 10 cas de la miliaire tuberculeuse. Le diagnostic était confirmé bactériologiquement chez la grande part des patients (84,09%). Les âges extrêmes et le sexe féminin étaient plus touchés par la tuberculose extrapulmonaire. La guérison était déclarée chez 35,12% des patients et elle était plus importante chez les malades à tuberculose pulmonaire par rapport aux patients à tuberculose extrapulmonaire (62,18% vs 0,37%; P<0,001). Les perdus de vue étaient plus importants parmi les patients touchés par la tuberculose pulmonaire (19,33% vs 10,81%; P<0,001). Le décès était survenu au presque même degrés d´importance chez les patients atteints de tuberculose pulmonaire et extrapulmonaire (2,52% vs 2,56%).

**Conclusion:**

des efforts, sur tous les plans, restent encore à déployer si le pays souhaite atteindre l´objectif de l´élimination de la tuberculose d´ici 2030.

## Abstract (en)

**Introduction:**

tuberculosis is a public health problem in Morocco. This study aims to examine the epidemiological profile as well as the evolutionary and diagnostic features of tuberculosis in Settat, Morocco.

**Methods:**

we conducted a retrospective descriptive and analytical study of data from the medical records of TB patients managed at the Diagnostic Centre for Tuberculosis and Respiratory Diseases in Settat, Morocco from 1^st^ January 2015 to 31 December 2019.

**Results:**

we identified 1270 cases of tuberculosis. Lung involvement was more common than extrapulmonary involvement. Ganglionic tuberculosis mainly occurred in patients with extrapulmonary tuberculosis. Severe forms included tuberculous meningitis (10 cases) and miliary tuberculosis (10 cases). Diagnosis was bacteriologically confirmed in the majority of patients (84.09%). Extreme ages and female sex were mainly affected by extrapulmonary tuberculosis. Recovery was reported in 35.12% of patients and its rate was higher in pulmonary TB patients than in extrapulmonary TB patients (62.18% vs 0.37%; P<0.001). Patients with pulmonary tuberculosis (19.33% vs 10.81%; P<0.001) were mainly lost to follow-up. Death occurred to almost the same extent in patients with pulmonary and extrapulmonary tuberculosis (2.52% vs. 2.56%).

**Conclusion:**

efforts on all fronts are still needed if the country is to achieve the goal of eliminating TB by 2030.

## Introduction

La tuberculose est une maladie infectieuse contagieuse due à une mycobactérie du complexe tuberculosis. Depuis toujours et dans toutes les civilisations humaines connues, la tuberculose n´a jamais cessé de faire des victimes. Encore aujourd´hui, elle représente un problème majeur de santé publique dans le monde. Le diagnostic est évoqué devant des données cliniques et radiologiques, mais la confirmation n´est que bactériologique et/ou histologique [1]. En dépit de l´amélioration des conditions de vie et l´existence d´un traitement efficace, la tuberculose reste l´une des maladies infectieuses et contagieuse les plus meurtrières, et fait actuellement partie des dix premières causes de mortalité dans le monde. L´Organisation mondiale de la Santé (OMS) a fixé pour objectif de l´éradiquer d´ici 2035 [2]. Chaque jour, près de 4 000 personnes perdent la vie à cause d´elle, soit près de 1,5 million de décès chaque année, et près de 28 000 personnes contractent cette maladie évitable et curable, soit près de 9 millions nouveau cas de tuberculose chaque année. Les efforts mondiaux de lutte contre la tuberculose ont permis de sauver environ 63 millions de vies depuis l´an 2000 [3]. Le Maroc est parmi les payes qui n´ont pas échappé au mal de la tuberculose. En 2019, le nombre de cas estimés par l´OMS était de 35 000 nouveaux cas et le nombre estimé de décès liés à la tuberculose était de 2.900 décès, soit un taux de mortalité spécifique de 8,1 pour 100.000 habitants. En 2020, le nombre de cas enregistrés était 29.018 cas, toutes formes confondues. Toujours en 2020, deux cent quarante cas co-infectés tuberculose-virus d´immunodéficience humaine (VIH) [4]. Dans le but de lutter contre cette maladie, le Maroc avait mis en place, depuis plusieurs décennies, un programme national de lutte antituberculeuse actif au niveau des établissements de soins de santé primaires de deuxième niveau, et dans les Centres de Diagnostic de la Tuberculose et des Maladies Respiratoires. Ce programme assure gratuitement toutes les prestations de diagnostic et de prise en charge des malades atteints de tuberculose. Aussi, dans le plan stratégique national de lutte contre la tuberculose pour la période 2018-2021, le Maroc vise à réduire le nombre de décès liés à la tuberculose de 40% en 2021 par rapport à l´année 2015.

Le présent travail s´est fixé pour objectifs d´étudier le profil épidémiologique, les aspects évolutifs et diagnostiques de la tuberculose pulmonaire comparée à la tuberculose extrapulmonaire dans la province de Settat incluse dans la région Casablanca-Settat au Maroc. En effet, à notre connaissance aucune étude sur de tel thème n´a été menée dans cette province. Dans la présente étude, nous retenons les définitions opérationnelles suivantes: **nouveau cas**: tout patient qui n´a jamais reçu de traitement antituberculeux ou qui a pris des médicaments antituberculeux pendant moins de quatre semaines consécutives. **Guérison**: un patient atteint de tuberculose pulmonaire chez qui l´affection a été confirmée bactériologiquement en début de traitement, présente des résultats négatifs (selon l´examen des frottis ou la mise en culture) au cours du dernier mois de traitement et au moins une fois auparavant. **Traitement terminé**: le patient tuberculeux a terminé le traitement sans signe d´échec, mais on ne dispose pas de données indiquant que les résultats de l´examen des frottis ou de la mise en culture ont été négatifs au cours du dernier mois de traitement et au moins une fois auparavant, soit parce que les tests n´ont pas été réalisés soit parce que les résultats ne sont pas disponibles. **Échec thérapeutique**: le patient tuberculeux continue de présenter des résultats positifs (selon l´examen des frottis ou la mise en culture) après cinq mois de traitement ou plus. **Décès**: le patient tuberculeux meurt pour une raison quelconque au cours du traitement ou avant de l´avoir commencé. **Perdu de vue**: le patient tuberculeux n´a pas entamé de traitement ou celui-ci a été interrompu pendant deux mois consécutifs ou plus. **Patient tuberculeux à statut inconnu pour le VIH**: désigne tout cas de tuberculose confirmé bactériologiquement ou diagnostiqué cliniquement pour lequel on ne possède pas de résultat de dépistage du VIH et pas non plus de données probantes indiquant qu´il suit des soins contre le VIH. Si le statut de ce patient par rapport au VIH est déterminé par la suite, il convient de le reclasser en conséquence.

## Méthodes

**Type et cadre de l´étude**: il s´agit d´une étude rétrospective descriptive et analytique, étalée sur une période de 5 ans (du 1^er^ janvier 2015 au 31 décembre 2019), qui a concerné une cohorte de patients tuberculeux. Le recueil des données est fait à partir des dossiers médicaux, registre de dépistage de VIH et registre d´enregistrement des patients suivis au centre de diagnostic de la tuberculose et des maladies respiratoires de Settat situé dans la région de Casablanca-Settat au Maroc. Ce centre desservie une population de 633575 selon le dernier recensement général de la population et de l´habitat mené au Maroc en 2014.

**Critères d´inclusion et d´exclusion des cas**: pour éviter les biais engendrés par l´échantillonnage, tous les cas de tuberculose diagnostiqués et déclarés pendant la période de l´étude étaient inclus.

**Analyse de données**: les variables étudiées sont représentées par les caractéristiques sociodémographiques (âge, sexe et lieu de résidence), le diagnostic topographique de la tuberculose, le statut sérologique vis-à-vis du VIH et le devenir du patient. L´analyse statistique était faite en utilisant le logiciel Epi-Info^TM^ version 7. L´analyse descriptive a permis de présenter les données générales. En analyse bivariée nous avons comparé, selon le site de la tuberculose, les proportions des variables qualitatives en utilisant le test de Chi-2 ou le test de Fisher exact selon les conditions d´application de chacun des tests. Le seuil de P inférieur à 0,05 était considéré comme significatif. Dans l´analyse comparative des patients à tuberculose pulmonaire et les patients à tuberculose extrapulmonaire, nous avons exclu la miliaire tuberculeuse considéré comme une forme grave de tuberculose due à la dissémination lymphohématogène des bacilles tuberculeux à partir d´une lésion focale (poumon ou autre organe).

**Considérations éthiques**: après avoir obtenu l´autorisation auprès des autorités sanitaires, la collecte des données était effectuée par l´infirmière, membre de l´équipe de travail. Durant toutes les étapes de l´étude, les données étaient traitées de façon anonyme et confidentielle et elles n´étaient utilisées que dans les objectifs assignés à cette étude.

## Résultats

**Figure 1:**
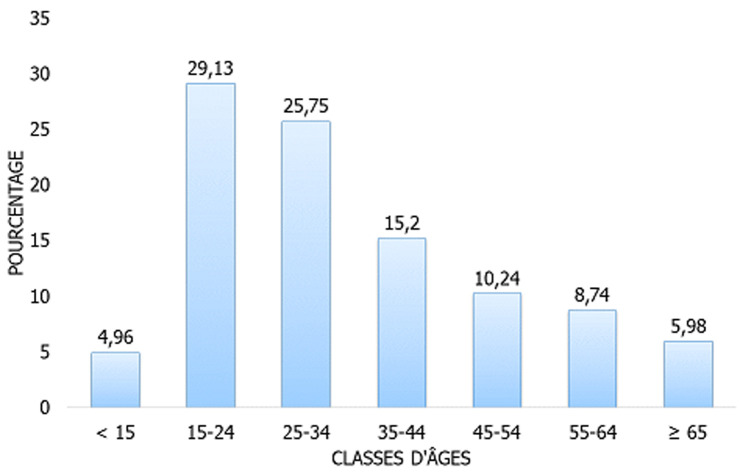
répartition des patients selon l’âge (N= 1270)

**Caractéristiques sociodémographiques des patients**: durant la période de l´étude, après avoir exclu 6 cas de primo-infection tuberculeuse, 1270 patients étaient enregistrés et inclus. Presque trois cinquièmes des patients étaient de sexe masculin (60,08%) soit un sex-ratio homme/femme de l´ordre de 1,50. Un peu plus de deux tiers de patients (68,66%) habitaient en milieu urbain. Plus de la moitié de nos enquêtés (54,88%) avait un âge compris entre 15 et 34 ans (Figure 1).

**Formes, aspects diagnostiques et sites anatomiques touchés par la tuberculose**: durant la période de l´étude, la tuberculose pulmonaire représentait plus de la moitié (56,22%) des cas de tuberculose (tuberculose pulmonaire à microscopie positive et tuberculose pulmonaire à microscopie négative et culture négative). Le diagnostic était confirmé bactériologiquement chez la grande part des patients (84,09%). Les localisations extra-pulmonaires étaient représentées essentiellement par l´atteinte ganglionnaire (18,03%) et pleurale (16,54%). La tuberculose pulmonaire à microscopie négative et culture négative représentait 7,28% des cas de tuberculose pulmonaire. Les formes graves de la tuberculose avaient affecté 20 patients, représentées par 10 cas de méningite tuberculeuse et 10 cas de la miliaire tuberculeuse, soit 1,57 % de l´ensemble des cas de tuberculose enregistrés toute formes confondues (Tableau 1).

**Tableau 1:** tuberculose (formes, aspects diagnostiques, sites anatomiques touchés, devenir des tuberculeux et statut vis-à-vis du VIH) (N= 1270)

Variables	Effectif	Pourcentage (%)
**Formes de tuberculose**		
Pulmonaire	714	56,22
Extrapulmonaire	546	42,99
Miliaire	10	0,79
**Tuberculose pulmonaire**		
*TPM+	662	92,72
**TPM-C-	52	7,28
**Diagnostic**		
Confirmé bactériologiquement	1068	84,09
Confirmé cliniquement	202	15,91
**Site anatomique touché**		
Poumons	714	56,22
Ganglions	229	18,03
Plèvres	210	16,54
Péritoine	38	2,99
Os	31	2,44
Méningite tuberculeuse	10	0,79
Uro-génitale	9	0,71
Peau	6	0,47
Autre	23	1,81
**Statut et issus**		
Guérison	446	35,12
Achèvement du traitement	476	37,48
Échec thérapeutique	8	0,63
Non évalué	90	7,09
Sous traitement	17	1,34
Perdu de vue	198	15,59
Décès	35	2,76
**Statut vis-à-vis du VIH**		
Statut inconnu	639	50,31
VIH-négatif	624	49,13
VIH-positif	7	0,55

* : Tuberculose pulmonaire à microscopie positive, **: Tuberculose pulmonaire à microscopie négative et culture négative

**Devenir des tuberculeux**: la guérison était déclarée chez plus d´un tiers des tuberculeux (35,12%). L´achèvement du traitement était aussi noté chez plus d´un tiers des patients (37,48%). Les perdus de vue et les décédés représentaient respectivement 15,59% et 2,76% des cas. L´échec thérapeutique était notifié chez 0,63% des cas (Tableau 1).

**Statut vis-à-vis du virus de l´immunodéficience humaine (VIH)**: le statut vis-à-vis du SIDA était inconnu chez presque la moitié des patients. Le SIDA était répertorié chez 0,55% des tuberculeux (Tableau 1).

**Facteurs associés aux formes de tuberculose**: la tuberculose pulmonaire avait touché les sujets du sexe masculin plus que les sujets de sexe féminins (67,46% vs 40,32% ; P<0,001) (Tableau 2). Les résidents en milieu urbain développaient davantage la tuberculose pulmonaire plus que les habitants du milieu rural (57,11% vs 55,70%). Toutefois cette différence n´était pas statistiquement significative (Tableau 2). Avoir un âge inférieur à 15 ans était associé à un risque d´attraper la tuberculose extrapulmonaire (79,37% vs 41,44% ; P<0,001) (Tableau 2). La tuberculose pulmonaire touchait la tranche d´âge comprise entre 55 et 64 ans plus que les autres tranches d´âges (P<0,001) (Tableau 2). La tuberculose extrapulmonaire affectait de façon significative les patients d´âges extrêmes (moins de 15 ans et plus de 65 ans) plus que les autres (P<0,001) (Tableau 2).

**Tableau 2:** facteurs associés aux formes de tuberculose (N= 1260)

Variables	Formes de tuberculose				P
	Pulmonaire		Extra-pulmonaire		
	Effectif	Pourcentage (%)	Effectif	Pourcentage (%)	
**Sexe**					
Masculin	512	67,46	247	32,54	**<0,001**
Féminin	202	40,32	299	59,68	
**Lieu de résidence**					
Urbain	494	57,11	371	42,89	NS*
Rural	220	55,70	175	44,30	
**Age <15 ans**					
Non	701	58,56	496	41,44	**<0,001**
Oui	13	20,63	50	79,37	
**âge ≥65 ans**					
Non	677	57,18	507	42,82	NS*
Oui	37	48,68	39	51,32	
**Tranches d´âges (ans)**					
<15	13	20,63	50	79,37	**<0,001**
15-24	210	57,22	157	42,78	
25-34	190	58,64	134	41,36	
35-44	114	59,69	77	40,31	
45-54	77	59,23	53	40,77	
55-64	73	66,97	36	33,03	

NS* : Non significative

**Association entre le devenir des patients et les formes de la tuberculose**: les perdus de vue étaient plus importants parmi les patients touchés par la tuberculose pulmonaire (19,33% vs 10,81%; P<0,001) (Tableau 3). Le décès était survenu au presque même degrés d´importance chez les patients atteints de tuberculose pulmonaire et extrapulmonaire (2,52% vs 2,56%) et d´ailleurs cette différence n´était pas statistiquement significative. À titre de comparaison avec les sujets touchés par la tuberculose extrapulmonaire, la guérison était plus importante chez les malades ayant attrapés la tuberculose pulmonaire (62,18% vs 0,37% ; P<0,001) (Tableau 3).

**Tableau 3:** association entre le devenir des patients et les formes de tuberculose (N= 1260)

Variables	Perdu de vue		P
	Oui: effectif (%)	Non: effectif (%)	
Pulmonaire	138 (19,33)	576 (80,67)	**<0,001**
Extra-pulmonaire	59 (10,81)	487 (89,19)	
	**Décès**		P
	Oui: effectif (%)	Non: effectif (%)	
Pulmonaire	18 (2,52)	696 (97,48)	NS*
Extra-pulmonaire	14 (2,56)	532 (97,44)	
	**Guérison**		P
	Oui: effectif (%)	Non: effectif (%)	
Pulmonaire	444 (62,18)	270 (37,82)	**<0,001**
Extra-pulmonaire	2 (0,37)	544 (99,63)	

NS* : Non significative

## Discussion

Pendant la période de l´étude étalée sur cinq ans (du 1^er^ janvier 2015 au 31 décembre 2019), nous avons répertorié 1270 cas de tuberculose. Dans notre étude, les hommes (60,08%) sont plus affectés que les femmes par la tuberculose toutes formes confondues (39,92%). Le sex-ratio était de 1,5 hommes pour une femme. Cette prédominance masculine était rapportée dans d´autres études [5,6]. Aussi, la tuberculose pulmonaire était prédominante parmi les hommes. Cette association a été aussi rapportée par plusieurs auteurs [7,8]. À l´encontre des masculins, les sujets de sexe féminins étaient plus touchés par la tuberculose extrapulmonaire. Ce résultat confirme les données de la littérature selon lesquelles les études montrent une prédominance féminine de la forme extrapulmonaire [9,10]. Si les bacilloscopies de crachats permettent de diagnostiquer la majorité des cas de tuberculose pulmonaire, la tuberculose pulmonaire à microscopie négative pose encore des difficultés diagnostiques et de prise en charge [11]. Dans la présente étude, le diagnostic était confirmé bactériologiquement chez la grande part des patients (84,09%). La tuberculose pulmonaire à microscopie négative et culture négative représentait 7,28% des cas de tuberculose pulmonaire.

Plus de la moitié des tuberculeux (54,88%) avait un âge compris entre 15 et 34 ans. Mais toutes les tranches d´âges étaient touchées ce qui renseigne sur le caractère contagieux de cette maladie. Les âges extrêmes (< 15 ans et ≥ 65 ans) étaient plus touchés par la tuberculose extrapulmonaire. Ce résultat corrobore les résultats trouvés par Ossalé Abacka *et al*. [10]. Cette prédominance des cas de tuberculose extrapulmonaire aux âges extrêmes pourrait être expliquée par les capacités immunitaires immatures chez les enfants et par l´immuno-sénescence chez les personnes âgées. Ceci rend ces personnes susceptibles de développer les tableaux cliniques frustres de la tuberculose, représentés en grande partie par les tuberculoses extrapulmonaires. La tuberculose pulmonaire touchait la tranche d´âge comprise entre 55 et 64 ans plus que les autres tranches d´âges. Dans l´étude menée par Ossalé Abacka *et al*. au centre antituberculeux d´Adjamé au Côte d'Ivoire c´était la tranche d´âge de 15 à 25 ans qui était plus touchée par cette forme de tuberculose [10]. Dans cette étude, l´atteinte pulmonaire était plus fréquente que l´atteinte extrapulmonaire. En effet, elle a concerné 56,22% contre 42,99% des cas. Ce résultat va dans le même sens que les résultats trouvés par d´autres auteurs [12]. Cette fréquence importante de la localisation pulmonaire pourrait être expliquée d´une part par le fait que le *Mycobacterium tuberculosis* présente une prédilection particulière pour le parenchyme pulmonaire et d´autre part par le sous-diagnostic des formes extrapulmonaires de la maladie, qui nécessite souvent des techniques invasives. En effet, dans les formes extrapulmonaires, les lésions étant paucibacillaires [13,14] et les prélèvements étant dans la majorité des cas difficiles à obtenir, le diagnostic souvent moins aisé et repose sur l´imagerie, les analyses microbiologiques, les examens anatomopathologiques et la clinique, qui restent trompeuses dans pas mal de cas, le diagnostic reste souvent purement présomptif [15]. Aussi, la morbi-mortalité des tuberculoses extrapulmonaires reste élevée notamment en cas d´atteinte multifocale [16]. Ainsi, nous pourrons perdre des personnes touchées par de telles formes avant même de les diagnostiquer. Encore, dans notre pays, la pratique de l´autopsie n´étant pas de règle, et de ce fait le nombre de cas de la tuberculose extrapulmonaire ne peut que rester faible.

En lien avec les formes extrapulmonaires, dans la présente étude l´atteinte ganglionnaire était prépondérante suivie de la localisation pleurale. Ce résultat confirme les données de la littérature selon lesquelles les ganglions et la plèvre sont, d´assez loin, les localisations extrapulmonaires les plus fréquentes [10,14,17]. Les formes graves de la tuberculose avaient affecté 20 patients, représentées par 10 cas de méningite tuberculeuse et 10 cas de la miliaire tuberculeuse, soit 1,57 % de l´ensemble des cas de tuberculose notifiés. Cette proportion reste légèrement faible par rapport à celle rapportée par Ossalé Abacka *et al*. qui ont trouvé 2,2 % au Côte d'Ivoire [10]. L´objectif global du traitement antituberculeux est de parvenir à une guérison sans rechute chez tous les patients, en interrompant la transmission de *Mycobacterium tuberculosis* et en empêchant l´acquisition (ou l´amplification) d´une résistance supplémentaire aux médicaments. La guérison était déclarée chez plus d´un tiers des tuberculeux (35,12%). Ce taux de guérison reste inférieur à celui rapporté par Assao Neino *et al*. à Niamy au Niger qui ont rapporté 74,6% [7]. Le taux de guérison faible trouvé par la présente étude pourrait être expliqué par la proportion importante des patient qui avaient terminé leurs traitement (37,48% soit plus d´un tiers des patients) pour lesquels nous ne disposons pas encore de données suffisantes pour déclarer leur guérison. La guérison était plus importante dans le groupe des malades ayant la tuberculose pulmonaire par rapport au groupe de patient ayant une tuberculose extrapulmonaire avec une différence statistiquement significative. L´échec thérapeutique était notifié chez 0,63% des cas. Ce résultat reste plus bas par rapport à 7% rapportée par Boushab *et al*. en Mauritanie [8] et à 2% rapportée par Nimagan *et al*. en Guinée Conakry [18]. Les perdus de vue et les décédés représentaient respectivement 15,59% et 2,76% des cas. Le taux des perdus de vue trouvé par la présente étude dépasse largement le taux de perdus de vue à l´échelon nationale qui était de l´ordre de 7,6% [4]. Aussi ce taux est supérieur à celui trouvé par Amadou *et al*. qui avaient rapporté 7,4% au Niger [5]. Comparativement au groupe de malades à tuberculose extrapulmonaire, le taux des perdus de vue était significativement plus important au sein du groupe des patients à tuberculose pulmonaire. Ce résultat est alarmant, en effet les pertes de vue au sein des malades à tuberculose pulmonaires va favoriser davantage la dissémination du pathogène.

Le nombre de décès, rapporté par la présente étude était de 35 cas. Tenant compte de la population totale desservie par le centre, lieu de l´étude, et qui s´élève à 633575 habitants, le taux de mortalité spécifique à la tuberculose était de 5,52 pour 100 000 habitants. Ce taux reste faible par rapport au taux national qui était de l´ordre de 8,1 pour 100 000 [4]. Le décès était survenu au presque même degrés d´importance chez les patients atteints de tuberculose pulmonaire et extrapulmonaire (2,52% vs 2,56%) et d´ailleurs cette différence n´était pas statistiquement significative. Toutefois, la mortalité rapportée dans la présente étude, était moindre par rapport à 22% des cas de décès trouvés par l´étude menée par Assao Neino *et al*. à Niamy au Niger [7]. Dans la présente étude, le statut vis-à-vis de VIH était négatif chez presque la moitié des patients et inconnu chez l´autre moitié. Ce résultat reste loin de l´objectif spécifique fixé par le plan stratégique national de prévention et contrôle tuberculose 2018-2021 qui vise, à l´horizon de 2021, de tester au VIH, 95% des malades tuberculeux notifiés et maintenir à 100% la proportion des malades co-infectés tuberculose/VIH mis sous traitement antirétroviral. Dans une étude menée sur une série de 117 cas de tuberculose, suivis au service des maladies infectieuses du centre hospitalier universitaire Ibn Rochd-Casablanca au Maroc, les auteurs ont trouvé une co-infection VIH confirmée chez 39,3% des patients [19]. Dans la présente étude, la co-infection tuberculose-VIH était répertorié chez seulement 0,55% des tuberculeux. Des efforts reste à déployer pour que cet examen soit systématique chez tous les tuberculeux. En effet, la tuberculose a émergé comme maladie opportuniste associée au SIDA et aussi la tuberculose augmente la réplication virale chez les personnes infectées par le VIH et accélère la progression de la maladie [19].

**Limites de l´étude**: certaines informations à savoir: suivi du poids, antécédents et profession manquent souvent. Aussi, le statut vis-à-vis de VIH est inconnu chez presque la moitié des enquêtés. Un nombre non négligeable de patients n´ont pas bénéficié de l´examen clinique pour évaluer leur état de santé et statuer sur leur état et issu.

## Conclusion

Sur une période de 5 ans, 1270 cas de tuberculose ont été notifiés au territoire desservi par le centre de diagnostic de la tuberculose et de maladies respiratoires de Settat. Le diagnostic était confirmé bactériologiquement chez la grande part des patients (84,09%). L´atteinte pulmonaire était plus fréquente que l´atteinte extrapulmonaire. Les sujets du sexe féminins étaient plus touchés par la tuberculose extrapulmonaire. Les âges extrêmes étaient associés à un risque d´attraper la tuberculose extrapulmonaire. En lien avec les formes extrapulmonaires, dans notre étude l´atteinte ganglionnaire était prépondérante. La guérison était plus importante dans le groupe des malades ayant la tuberculose pulmonaire par rapport au groupe de patient ayant une tuberculose extrapulmonaire avec une différence statistiquement significative. L´échec thérapeutique était notifié chez 0,63% des cas. Le décès était survenu au presque même degrés d´importance chez les patients atteints de tuberculose pulmonaire et extrapulmonaire (2,52% vs 2,56%). Sans l´adoption d´une approche intégrée mettant la lutte antituberculeuse au cœur de toutes les politiques, l´élimination de la tuberculose dans notre pays d´ici 2030 reste un défi difficile à atteindre.

### Etat des connaissances sur le sujet

- *Au Maroc, la tuberculose représente un véritable problème de santé publique;*
- *Le patient tuberculeux, contagieux et perdu de vue, ensemence son entourage;*
- *La non-observance thérapeutique entraine la sélection et le maintien des souches mycobactériennes multi-résistantes*.

### Contribution de notre étude à la connaissance

- *La présente étude dresse un état des lieux en lien avec la tuberculose humaine à la province de Settat au Maroc (première étude dans cette zone);*
- *Les perdus de vue, et donc la non-observance thérapeutique, sont important parmi les patients atteints de tuberculose pulmonaire, ceci va contribuer dans l´augmentation du risque d´avoir plus de cas de tuberculose multirésistante dans l´avenir;*
- *L´analyse de la situation épidémiologique de la tuberculose au niveau de cette province montre que la tuberculose restera un problème de santé publique au Maroc*.
